# Health Informatics—Ambitions and Purpose

**DOI:** 10.3389/fdgth.2019.00002

**Published:** 2019-12-23

**Authors:** Uwe Aickelin, Wendy W. Chapman, Graeme K. Hart

**Affiliations:** School of Computing and Information Systems, The University of Melbourne, Melbourne, VIC, Australia

**Keywords:** health informatics, health informatics applications, health informatics and information systems, grand challenge, digital health

The current transformation of the digital health landscape is not only technological, it's also social, cognitive, and political, with the end goal participatory health—a partnership with digital devices collecting data and generating insights with new models of care evolving through partnerships of clinicians, patients, and carers.

Most people are likely aware of the enormous challenges facing the world to deliver equitable, affordable healthcare to a world population that is growing, aging, undergoing increasing urbanisation, and suffering more chronic preventable diseases. Consumer expectations of their health practitioners are growing, and the cost of devices and medications are constantly rising. Increasing antibiotic resistance may challenge the very existence of hospital based care. Added to this, the looming impacts of climate change on communicable disease patterns, social disruption, and migration patterns together with the cost of climate mitigation will reduce the financial pool available for health care (1).

How then can we use new technologies to keep up, or better yet, get in front of the curve, to reduce cost, improve safety, increase equity of access, reduce the burden on limited hospital facilities, and increase the overall health and well-being of the community at large?

Regardless of the social or funding model, reducing avoidable complications through evidence based, personalised, connected care is essential. Increasing patient focus and patient control are necessary elements of the individual responsibility for health and wellness. Managing social determinants of health in our population also has the potential for increasing the return on investment of our health expenditure (2).

For those of us working in health, the current convergence of wireless communications, miniaturisation of physiological sensors, Electronic Medical/Health Records (EMRs), mobile computing interfaces (including smartphones), advanced analytics, personalised medicine, and connected data repositories provides a source of optimism that dramatic transformation and improved cost effectiveness can be achieved in the foreseeable future. Examples include prediction return to work after rehabilitation or understanding factors that may lead to complications in health procedures (3, 4).

There remain significant hurdles to overcome in our quest. System disruption from consumer wearable devices improves consumer-directed care, but this increasing independence may paradoxically isolate the patient from their care providers. Connection between data systems is dramatically increasing, but data aggregation and analysis is still limited by different vendor EMR data structures, competing terminologies and ontologies, jurisdictional data sharing, and privacy legislative differences. Telehealth has improved access to many forms of clinical support, however rural and remote communities still suffer from physical isolation and access to interventional services.

As acute hospitals reduce the need for inpatient care, do we have the trained workforce to care for patients at home? Will telemedicine, remote monitoring, GP and community sectors be able to assume the load? How will the economic and regulatory enablers be established to promote appropriate outcomes? What then will be the role of digital technologies in identifying and supporting patients most at risk?

As the EMR becomes more embedded, some jurisdictions have discovered that unparalleled ability to capture data including repetitive clinical documentation for compliance purposes may reduce the capacity of the system overall through staff burnout and clinical resistance.

A necessary step in improvement is understanding that our most dramatic increase in capability is derived from our closure of the data cycle. The advent of EMR, connected Acute and family medicine—General Practice data systems, real-time pathology and radiology results, device-generated physiology and mobility data enables rapid cycle individual outcomes assessment and population level reporting, as well as algorithmic notification of early disease markers, medication safety, conformance with evidence-based care, and pragmatic assessment of changes in health system delivery. These enhancements can collectively be seen as Learning Health Systems and will be essential in providing safe, effective, and evidence based care, regardless of the economic and political framework.

At a research level, we now have numerous examples of machine learning and artificial intelligence impacting on the routine clinical diagnostic process. However, these algorithms must become trusted partners in a sustainable health system. The algorithms must be validated prior to implementation, embedded in the workflow and enhance the operational capacity of the human sponsors. Re-tasking and supporting human resources in the most effective manner may enable more humanistic interactions between clinicians and patients, allow development of newer techniques or simply just enable us to keep up with the ever increasing workload.

Regardless of which scenarios play out in the future, the social dimensions of these changes will be far reaching, and are just as worthy of study as the technical achievements themselves.

## Health Informatics as a Public Good

Health Informatics is the practice of acquiring, studying and managing health data, and applying medical concepts in conjunction with information technology systems to help clinicians provide better healthcare. We believe useful insights can be gained by viewing Health Informatics in general, and electronic healthcare records in particular, as a “public good.” A public good is any service or resource that cannot be withheld from an individual due to inalienable characteristics relating to citizens' rights (5). Examples of public good resources include city parks, street lighting or freeways, which are funded by the state but available to all.

Economists have extensively studied public goods and developed so-called public goods games to simulate and understand people's behaviour (6). It has been observed through such studies that players adjust their contribution according to the behaviour of other players. For instance, an initial willingness for contribution might change due to learning about other players' contribution behaviour. Broadly, players can be classified into four types based on their aggregate contributions (6):

- Unconditional Contributors: Players who contribute regardless of the behaviour of other players.
- Conditional Co-operator: Players who show more willingness to contribute when other players contribute more.
- Free Riders: Players who do not contribute to the project regardless of other players' contribution status.
- Triangle Contributors: Players whose contribution rises to a point then starts to decline in relation to other players' contributions.

Looking at Health Informatics through this lens opens up new perspectives on how to address problems such as data handling challenges. For instance, using Artificial Intelligence methodologies in clinical decision support raises issues such as explainability (why), interpretability (how), and trust (who). By understanding we are dealing with a public good, we learn that we need to cooperate rather than compete. Therefore, neither clinicians nor patients can simply be seen as consumers of health information systems, they must be co-creators.

## Workforce Development Required for the Future

We need a much more extensive program for workforce development and education than currently exists to reach a digital transformation of health.

Targeted students should include:

- Clinicians providing patient care.
- Data scientists, analysts and IT professionals working in hospitals, insurance companies, government and the health tech industry.
- Students enrolled in professional programs in the health field, such as medicine, nursing, physiotherapy, nutrition, etc.
- Students enrolled in degree-granting programs such as informatics, computer science, mathematics, psychology, and economics who are interested in applying their knowledge and skills in the health domain.
- Clinician scientists who combine research with practice to effect change in health care delivery.
- CEOs and other executives in health care delivery systems.
- Politicians making regulatory decisions.

To reach such a broad group of students we require layers of resources that not only vary in content, but also in the amount of depth that is provided and in teaching methods. Resources including:

- Online lectures, interviews, and case studies that can inspire and motivate.
- Hands-on skill building activities that provide experience wrangling, analysing, and interpreting health data sets and demonstrating how to transform data into actionable insights.
- Interaction with electronic medical records, business intelligence tools, and AI applications that provide experience with systems used now and in the future.
- Sociotechnical activities such as interactive simulations, observations, and individual or focus group interviews that provide insight into the social and cognitive aspects of digital health technology embedded in health settings.
- Intensive mentoring programs that are necessary for training in digital health and informatics research.

Those who have taught in this space are painfully familiar with the benefits and challenges of a multidisciplinary classroom. Successful training of clinical trainees requires skill development in data management, programming, and information modelling. Whereas, students without a clinical background need knowledge of the healthcare environment, medical terminology and physiology, and healthcare economics. These topics are best integrated into existing training activities so they are not considered add-ons to already full programs.

Considering these points, it becomes obvious that we need a two-way “conversion” or indeed “confluence,” from health to IT and vice versa. Thus, ideal programmes are those based on Computer Science or Information Systems, but with a specialisation in health or medicine, for example a Master of Information Systems with a Health Specialisation. In such degrees it is crucial that students have opportunities for industry-based learning in the IT and health sectors and that the curriculum is aligned with national and international guidelines of the foundational discipline of Health Informatics. Ideally, elective streams are available in areas such as Information Systems Project and Change Management, IT Service Provision, Business Analytics, IT Innovation and Interaction Design, and Spatial Information.

Extensive infrastructure is required to support hands-on learning, involvement in clinical settings, and participation in research or quality improvement topics. If Health Informatics can be viewed as a public good, so can workforce development and education in this space. However, with current incentive structures, it isn't clear whether one or a few entities could host and manage materials made and consumed by diverse players. We need more innovation in informatics education delivery.

There are some early efforts to make educational resources modular for use and re-use in micro-credentialing, professional development, and coordinated programs of study. However, there is still much to be done to align incentives of trainees, universities, professional societies, and industry in a student-centred way so that everyone has access to the learning they need.

## How Will This Journal Contribute

This section is looking for interdisciplinary high-quality submissions that integrate information technology with health science. Importantly, we are not only encouraging traditional academic contributions (such as data-driven or discovery-led articles), but we are also eager to receive real-world case studies and review papers written by practitioners. We believe this is essential to illuminate the barriers and catalysts to successful adoption, innovation, development, implementation, and evaluation in such healthcare technologies and applications. Furthermore, we welcome submissions about education and workforce development, social and ethical implications of digital health, and economic analysis.

This approach to article curation is supported by the distinct reviewing and epitomal systems employed by Frontiers. Rather than the typical adversarial “authors vs. referees” approach, we operate an open review and editorial process where we collaboratively, as a team, improve, and perfect articles with the common aim of making the journal the best it can be.

## Author Contributions

All authors listed have made a substantial, direct and intellectual contribution to the work, and approved it for publication.

### Conflict of Interest

The authors declare that the research was conducted in the absence of any commercial or financial relationships that could be construed as a potential conflict of interest.
